# *Plasmodium* attenuation: connecting the dots between early immune responses and malaria disease severity

**DOI:** 10.3389/fmicb.2014.00658

**Published:** 2014-12-02

**Authors:** Priyanka Fernandes, Roland Frank, Matthew D. Lewis, Ann-Kristin Mueller

**Affiliations:** Parasitology Unit, Centre of Infectious Diseases, University Hospital HeidelbergHeidelberg, Germany

**Keywords:** malaria, attenuation, experimental cerebral malaria, early immune response

## Abstract

Sterile attenuation of *Plasmodium* parasites at the liver-stage either by irradiation or genetic modification, or at the blood-stage by chemoprophylaxis, has been shown to induce immune responses that can protect against subsequent wild-type infection. However, following certain interventions, parasite attenuation can be incomplete or non-sterile. Instead parasites are rendered developmentally stunted but still capable of establishing an acute infection. In experiments involving *Plasmodium berghei* ANKA, a model of experimental cerebral malaria, it has been observed that several forms of attenuated parasites do not induce cerebral pathology. In this perspective we collect evidence from studies on murine malaria in particular, and attempt to “connect the dots” between early immune responses and protection from severe cerebral disease, highlighting potential parallels to human infection.

## INTRODUCTION

Radiation-attenuated sporozoites, genetically attenuated parasites and sporozoites administered under chemoprophylaxis have all been shown to successfully induce long-lasting sterile protection against malaria infection, in both animal models and humans (Nussenzweig et al., 1967; Hoffman et al., 2002; Belnoue et al., 2004; Mueller et al., 2005; Roestenberg et al., 2009; Butler et al., 2011). Sterile protection is characterized by the elimination of the parasite through the pre-erythrocytic immune response before it is able to establish a blood infection, that can be detected microscopically and cause pathology. In contrast, protection from severe cerebral pathology does not necessitate the complete elimination of the parasite from the host. Instead, parasites transition from the liver into the blood but do not cause severe cerebral pathology.

This perspective focusses on attenuated, non-sterile infections, particularly those from studies on murine malaria, that result either intentionally or unintentionally from previous interventions such as immunization or genetic manipulation of parasite lines. Such interventions eventually limit or alleviate the murine severe disease outcome, experimental cerebral malaria (ECM). Moreover, we summarize evidence from both published and unpublished observations that suggest a critical role for early immune responses in influencing the development of cerebral pathology.

Experimental cerebral malaria develops when susceptible mouse strains are infected with specific strains of *Plasmodium.* For example, infection of C57BL/6 mice with *Plasmodium berghei* ANKA (*Pb*A) generates a severe cerebral syndrome generally considered to be analogous to human cerebral malaria (de Souza and Riley, 2002). Descriptions of ECM immunopathogenesis are multifaceted and largely hypothetical, involving an intricate series of interactions in space and time between both host and pathogen, but generally start from the onset of blood-stage infection (Renia et al., 2006). ECM pathology is generally marked by ataxia, fitting, coma, and eventually death (de Souza and Riley, 2002). Although subject to some debate (Carvalho, 2010; White et al., 2010; Craig et al., 2012), the close correlation between mice and humans in terms of immune responses and neuropathological processes (Hau and Van Hoosier, 2005; de Souza et al., 2010) has made ECM a generally accepted, if sometimes disputed, model (Hunt et al., 2010; Riley et al., 2010; Langhorne et al., 2011).

Few studies to date have addressed the potential for host immune responses directed against either the liver-stages or early blood-stages to modify the immunopathogenesis of ECM. Our experience is that the apparent absence of ECM following parasite manipulation is often considered irrelevant and hence not fully examined yet, for example during the functional characterization process of a transgenic parasite. Indeed, the absence of ECM following numerous experimental conditions is an accepted, anecdotally recounted but rarely published phenomenon.

In an attempt to shed light on a generally disregarded aspect of murine malaria, we have outlined several examples of malaria infection that share the same outcome of protection from severe cerebral complications, but in all probability do not share the same protective mechanism. It is our hope that highlighting and cataloging examples of this generally disregarded phenomenon will both draw attention to the field and help piece together potential critical factors involved in protection against cerebral malaria.

## INCOMPLETE PARASITE ATTENUATION AT THE LIVER-STAGE

Haussig et al. (2011) recently observed that targeting the apicoplast by disruption of a *Plasmodium*-specific protein that plays a role in liver merozoite formation (PALM) affected liver-stage development and the subsequent onset of blood-stage parasitemia. 30% of the *palm*(-)-immunized mice became patent following a delay of up to 4 days, of which the majority did not develop cerebral pathology (Haussig et al., 2011).

Another approach described an experimental vaccination regime consisting of sporozoite immunization applied concomitantly with either azithromycin or clindamycin drug cover. This permits full development of the malarial liver-stage, but inhibits the inheritance and biogenesis of the apicoplast, thus preventing the onset of blood-stage infection. While sterile protection was found to depend on IFN-γ producing CD8^+^ T cells that exclusively targeted the intra-hepatic stages, it was dose-dependent and a reduction in the sporozoite numbers used for immunization led to breakthrough infections. However, all mice that developed blood-stage infecion featured a delay in the onset of patency and were apparently protected from ECM (Friesen et al., 2010).

Further it is known that sterile protection conferred by immunization under chloroquine (CQ) cover relies on a critical threshold of intra-hepatic parasites (Nganou-Makamdop et al., 2012). Comparable to Friesen et al. we have observed that a reduction in the sporozoite numbers used for immunization under CQ cover, does not confer sterile protection against a wild type infection, but delays the onset of blood-stages and protects against ECM (Pfeil et al., unpublished).

While the mechanism behind protection against severe cerebral symptoms in the models described above still remains elusive, preliminary data from another experiment suggests a role for an altered host immune response in the modulation of ECM outcome. Incomplete attenuation of *Pb*A parasites achieved via sub-therapeutic administration of a liver acting anti-malarial substance lead to the suppression of intrahepatic development, a delay in prepatency and subsequent abrogation of cerebral pathology. This effect was supported by a robust host immune environment involving a Th1 response and early T-cell activation in both liver and spleen (Lewis et al., unpublished).

A common motif between these observations is developmental impairment or attenuation during the transition from liver to the intraerythrocytic phase of the malaria parasite. It is conceivable that this altered transition results in a slow trickle of parasites into the bloodstream. How exactly this slow onset of blood-stage parasitemia modulates the immune response in a way that severe disease is prevented, remains unknown.

## INCOMPLETE PARASITE ATTENUATION AT THE BLOOD-STAGE

The notion that parasite growth kinetics in the blood can be linked to cerebral malaria, while tenuous, is not entirely novel. In fact, several murine studies have documented early growth defects in the blood that caused an altered disease outcome. One such example was the oral administration of trioxane T-10 thioacetals to C57BL/6 mice 24 h after infection with *Pb*A-infected erythrocytes, which completely abrogated ECM in treated mice (Jacobine et al., 2012). Deletion of certain non-essential blood-stage antigens also achieves the same result. For example *P. berghei* parasites lacking the endogenous merozoite surface protein 7 (MSP7) remain viable, but are impaired in their multiplication rates in the blood (Tewari et al., 2005). A minor delay in parasite development *in vivo*, was attributed to enhanced reticulocyte preference but was sufficient to ablate ECM in C57BL/6 mice (Tewari et al., 2005; Spaccapelo et al., 2011). Similar virulence-attenuated phenotypes were also observed in experiments with parasites lacking plasmepsin 4 (*Δpm4*) or a component of the PTEX, thioredoxin-2 (TRX2) *ΔPbTRX-2*. Protection from ECM in the case of *Δpm4* parasites was associated with a growth defect in the blood (Spaccapelo et al., 2010). While *ΔPbTRX2* mutants displayed a marked delay in parasitemia resulting in abrogation of ECM in the majority of mice, variations in virulence were observed between *ΔPbTRX2* clones, which the authors hypothesized resulted from differences in the number of times the clones had been passaged (Matthews et al., 2013).

An inference drawn from the examples above suggests that chemical or genetic methods of attenuation modify parasite growth in the blood in a way that differs from a natural infection. This form of attenuation could potentially stall parasite development, thereby reducing the burden of viable parasites and the ensuing immunopathogenesis, thus resulting in the abrogation of ECM.

## PARASITE ATTENUATION AND CLINICAL OUTCOME IN HUMANS

Although not directly comparable to the examples described above, similar observations have also been reported from human clinical trials. The partially protective effect against clinical and severe disease following immunization of individuals with the leading malaria vaccine candidate RTS,S represents a good example. The fact that a vaccine against pre-erythrocytic stages confers protection against severe malaria was suggested to stem from vaccine-induced immune responses that reduced the number of liver-stage parasites after natural infection. Such partial pre-erythrocytic immunity may result in the “leakage” of small numbers of parasites. This slow onset of blood-stage parasitemia might increase the time frame required to establish innate and adaptive immune responses that inhibit blood-stage growth and consequently limit severe disease (Guinovart et al., 2009). In a similar setting, long-term reduction in the risk of clinical malaria in Tanzanian children was observed following intermittent preventive treatment with the antimalarial sulfadoxine-pyrimethamine (SP). It was proposed that the long half-life and possibly anti-liver-stage acting properties of SP lead to low-dose blood-stage infections that effectively induce prolonged protection from clinical malaria (Schellenberg et al., 2001; Greenwood, 2007; Sutherland et al., 2007). Such clinical studies and many others that test vaccine efficacy or antimalarial drug potency, however, lack a detailed understanding of the dowmstream effects on human cerebral malaria.

## EARLY IMMUNE RESPONSES AND EVENTS THAT MAY AFFECT DOWNSTREAM IMMUNOPATHOGENESIS

Early immune responses and particularly elements and mechanisms of the innate immune system can influence downstream effector responses and consequently disease outcome (O’Garra and Murphy, 1994; Jankovic et al., 2001; Mitchell et al., 2005).

*In vitro* observations with *P. falciparum* and also murine studies have shown that infected red blood cells and parasite moieties such as glycosylphosphatidylinositol (GPI) and hemozoin can trigger innate pathways of the immune system, primarily through toll-like receptor signaling (Schofield et al., 1996; Coban et al., 2005). A study in the rodent model, that was published in 2007 identified TLR-2, -9 and MyD88-dependent signaling as mediators of ECM (Coban et al., 2007). However, subsequent studies showed that TLR-deficient mice still succumbed to ECM (Togbe et al., 2007; Lepenies et al., 2008), thus pointing out a controversial role for TLRs in the development of cerebral pathology.

Nevertheless, other components of the innate immune system have been implicated in the induction of ECM (Hansen et al., 2003, 2007; Maglinao et al., 2013; Palomo et al., 2013). For instance, Hansen et al. (2003) showed that susceptibility or resistance to ECM was dependent on CD1d-restricted NKT cells that modulated Th1/Th2 polarization. A subsequent study showed that NK cell depletion negated T cell recruitment to the brains of ECM-affected mice thus substantiating a role for NK cells in the regulation of adaptive immune responses that influence cerebral pathology (Hansen et al., 2007). Additionally, NK cells and γδ T cells, are also known as early sources of IFN-γ that could enhance parasite clearance mechanisms (Seixas and Langhorne, 1999; Artavanis-Tsakonas and Riley, 2002; Ing and Stevenson, 2009; Inoue et al., 2013).

Indeed, there is evidence that very early inflammatory responses are capable of altering downstream immunopathogenesis in a manner that involves CD8^+^ T cells and IFN-γ (De Souza et al., 1997; Mitchell et al., 2005; Lewis et al., unpublished). ECM-susceptible mice, co-infected with *Pb*A and *Pb*K173 are completely protected from ECM and this protection was found to be associated with increased IFN-γ in the blood at 24 h post-infection and an increase in transcriptional abundance of IFN-γ, IL-10 and IL-12 in both the liver and spleen (Mitchell et al., 2005). In this model early production of IFN-γ was attributed predominantly to CD8^+^ T cells that are known for their ability to rapidly produce this cytokine in a non-antigen-specific manner thereby contributing to innate immunity, e.g., in the early phase of bacterial infections (Berg et al., 2002, 2003; Kambayashi et al., 2003).

This is perhaps contradictory to the received wisdom that ECM is Th1 in nature and responsibility for pathology lies with IFN-γ, CD8^+^ T cells (de Souza and Riley, 2002) and the Th1-biased C57BL/6 mouse (Locksley et al., 1987). The answer partly lies with the opposing roles of IFN-γ or TNF-α depending on the time of their production during infection, i.e., early expression correlates with protection from ECM while later expression promotes ECM (Grau et al., 1989; de Souza and Riley, 2002; Mitchell et al., 2005). One could speculate that an early inflammatory peak disrupts the delicate balance required for ECM immunopathogenesis. A possible explanation is that parasite elimination mechanisms are enhanced, thus preventing the critical antigen threshold required for the onset of immunopathogenesis (Howland et al., 2013).

Alternatively, early inflammatory responses could also induce early production of anti-inflammatory cytokines such as IL-10, a critical regulator in ECM immunopathogenesis (Kossodo et al., 1997; Couper et al., 2008; Niikura et al., 2010). Our preliminary data also indicates that an early acute systemic inflammation may provoke the production of IL-10 (Lewis et al., unpublished). IL-10 may then alleviate CD8^+^ T cell activation, proliferation and downregulate the expression of adhesion molecules on the vascular endothelium (Renia et al., 2006). Thus the timing and localization of the production of pro- and anti-inflammatory cytokines is crucial to the development of cerebral immunopathogenesis.

Although we are limited in our understanding of the impact of early immune responses on the development of cerebral malaria in humans, studies from mouse models have suggested that an ability to control the initial parasitemia permits the development of adaptive immune responses that support an early inflammatory response and enhance parasite clearance (Meding and Langhorne, 1991; Mohan et al., 1997; van der Heyde et al., 1997; Su and Stevenson, 2000). An early inflammatory response could in turn dampen the immunopathology that otherwise prevails during a natural infection.

Since ECM is likely caused by a series of immunopathogenic mechanisms that are interrelated but not necessarily sequential or reliant upon each other, the disruption of one mechanism in a given model may not necessarily translate into the same outcome in another.

Nevertheless, we propose the following mechanisms by which growth impairment might play a role in the abrogation of ECM.

### A GROWTH DEFECT MAY AFFECT SEQUESTRATION IN PERIPHERAL ORGANS

Shortly after the onset of blood-stage infection, parasitized erythrocytes adhere to the peripheral tissues (Beeson et al., 2001), inducing the activation of monocytes, neutrophils, and DCs (Renia et al., 2006). The adherence of parasitized erythrocytes to the vascular endothelium has been shown to induce chemokine secretion and provoke an “activated” state in the brain endothelium. Leukocytes and parasitized erythrocytes bound to the endothelium interfere with the circulation and produce cytotoxic molecules. This damages the blood-brain-barrier and causes hemorrhages and oedema (de Souza et al., 2010). A blood-stage growth “defect” or “modification” may alter the kinetics of the replicating parasite, thereby altering the localization, severity or timing of parasite sequestration. In turn, this may modify the induced innate immune response.

### GROWTH KINETICS COULD ALTER ANTIGEN PRESENTATION

Given the shared antigen repertoire between liver and blood-stage parasites (Belnoue et al., 2008; Tarun et al., 2008), it is conceivable that altered or possibly prolonged presentation of shared antigens by late liver-stages directly influences the early immune response against erythrocytic stages, eventually altering disease outcome. Additionally, the timing and localization of parasite sequestration may also impact upon the adaptive immune response to malaria infection. CD8^+^ T cells are a critical requirement for cerebral pathology and disruption of their chemotaxis to the brain can protect against cerebral pathology (Renia et al., 2006). A recent publication indicated that T cells specific for a malarial glideosome-associated protein may be responsible for ECM pathology due to cross-presentation of parasite antigen by the brain microvessels (Howland et al., 2013). Activated endothelial cells are capable of presenting antigen in an MHC-I context (Pober and Cotran, 1991) and are likely to phagocytose sequestered parasite material for presentation of antigen to CD8^+^ T cells (Renia et al., 2006). The authors also demonstrated that the number of antigen-specific T cells does not increase in ECM models or upon the induction of cerebral pathology. Instead, the degree of cross-presentation of parasite moieties by the activated brain endothelium is reduced (Howland et al., 2013). A reduction in parasite sequestration may therefore reduce the priming of the immune events that would otherwise, like a line of dominoes, lead to the induction of cerebral pathology.

### PRIMING IN THE SPLEEN COULD BE MODIFIED BY GROWTH IMPAIRMENT

As the organ responsible for blood filtration, the spleen is likely to be responsible for the priming and activation of T cells that subsequently migrate to the brain (Renia et al., 2006). Indeed, splenectomised mice infected with low doses of *Pb*K173 do not develop cerebral symptoms (Curfs et al., 1989; Hermsen et al., 1998). Activation occurs at an early timepoint post-infection and it is conceivable that modification of parasite growth kinetics may reduce the exposure of parasite moieties to macrophages and dendritic cells within the splenic tissues, modifying the phagocytosis, processing and presentation of antigen. It is known that CD11c^hi^CD8^+^ dendritic cells are responsible for ECM immunopathogenesis (Piva et al., 2012) by phagocytosing dying cells and processing antigen in an MHC-I restricted manner (den Haan et al., 2000). Disrupting this process may modify the priming of lymphocytes which, in turn, may reduce systemic inflammation and endothelial cell activation.

### REGULATORY T CELLS

Although the role of regulatory T cells in malaria is controversial, it is conceivable that they play a role in the protection we observe in some models. ECM can be ablated in normal *Pb*A infection by the expansion of regulatory T cells (Haque et al., 2010). One possibility is that regulatory T cells temper the pro-inflammatory response (Riley et al., 2006), which is a key factor in the development of cerebral malaria. In fact, concomitant infection of mice with *Schistosoma japonicum* and *P. berghei* reduces ECM mortality by promoting a Th2 response that is supported by proliferating Tregs (Wang et al., 2013). Interestingly, protection from the severe symptoms of malaria in *P. falciparum* is also associated with the expansion of CD4^+^CD45RO^+^FOXP3^-^ regulatory T cells (Walther et al., 2009).

## CONCLUSION

The examples elaborated above substantiate a crucial role of early immune responses in influencing the immunopathogenesis of ECM. While a clear distinction cannot be drawn between the responses toward late liver-stages and those toward the early blood-stages, they both seem to exert an effect on the onset of parasitemia. Attenuated infections could serve as tools to improve our understanding of the mechanisms by which early immune responses regulate downstream adaptive immunity and consequently cerebral pathology. Elucidating these mechanisms could help refine future intervention strategies.

## Conflict of Interest Statement

The authors declare that the research was conducted in the absence of any commercial or financial relationships that could be construed as a potential conflict of interest.
